# EXAMINING SUBJECTIVE BURDEN AMONG SPOUSAL CAREGIVERS OF PARTNERS WITH DEMENTIA: THE IMPACT ON MARITAL QUALITY

**DOI:** 10.1093/geroni/igac059.1769

**Published:** 2022-12-20

**Authors:** Jeremy Yorgason, Avalon White

**Affiliations:** Brigham Young University, Provo, Utah, United States; Brigham Young University, Provo, Utah, United States

## Abstract

Although sometimes caregiving is associated with closeness in marriage, at other times the stresses may affect marriage relationships negatively. In the current study we explore how caring for a partner with dementia, and subjective burden are associated with marital quality. We further explore how dementia care and subjective burden might interact (suggesting a pileup of stress) to affect marital quality. Using data from 1,066 spousal caregivers that participated in the NSOC study and their corresponding care recipients from the NHATS study, the current analysis explored cross-sectional associations between spousal caregiving (primary vs. Secondary caregiver, subjective burden, gender, age, education) and partner care recipient characteristics (dementia classification, household income) predicting positive and negative marital quality. Results suggested that dementia classification and subjective burden were associated with lower positive marital quality and higher negative marital quality. The relationship between subjective burden and positive marital quality was moderated by whether or not the care recipient had dementia. Specifically, when dementia was present, the negative association between subjective burden and positive marital quality was stronger. Spousal caregivers often carry substantial burden to help their partners with activities of daily living. Care provision can alter the marriage relationship in negative ways when perceived burden is high. The current study suggests that the negative association between burden and positive marital quality is even stronger when caring for a spouse with dementia. Gender differences need further exploration, as well as how these patterns play out in early vs. later stages of dementia and across time.

